# Early chest x-ray severity assessed by the MBrixia score is associated with mortality in hospitalized patients with COVID-19 pneumonia

**DOI:** 10.1038/s41598-026-39285-x

**Published:** 2026-02-11

**Authors:** Christian Møller Jensen, Ramtin Z. Marandi, Junia C. Costa, Dennis Zetner, Siv Mari Sollien Berger, Carsten U. Niemann, Sisse R. Ostrowski, Joanne Reekie, Birgit Holten, Anna Kalhauge, Bruno Ledergerber, Jens Lundgren, Marie Helleberg, Kasper S. Moestrup

**Affiliations:** 1 Centre of Excellence for Health, Immunity and Infections (CHIP), Rigshospitalet, Blegdamsvej 9, Copenhagen Ø, 2100 Denmark; 2 Department of Diagnostic Radiology, Rigshospitalet, Blegdamsvej 9, Copenhagen Ø, 2100 Denmark; 3 Department of Haematology, Rigshospitalet, Blegdamsvej 9, Copenhagen Ø, 2100 Denmark; 4 Danish Cancer Society, Strandboulevarden 49, Copenhagen Ø, 2100 Denmark; 5 Department of Clinical Immunology, Rigshospitalet, Blegdamsvej 9, Copenhagen Ø, 2100 Denmark; 6 Department of Infectious Diseases, Rigshospitalet, Blegdamsvej 9, Copenhagen Ø, 2100 Denmark; 7 Department of Clinical Medicine, University of Copenhagen, Blegdamsvej 3B, Copenhagen N, 2200 Denmark

**Keywords:** COVID-19, Chest x-ray score, Mortality, Respiratory support, Diseases, Medical research, Risk factors

## Abstract

Chest x-ray (CXR) severity scores, such as the MBrixia score, are associated with COVID-19 severity, but most have not been validated after the introduction of antivirals and dexamethasone as standard of care. The MBrixia score’s association with mortality and the interobserver variability among radiologists remain unclear. Adults hospitalized with COVID-19 at a tertiary center in Denmark between June 15, 2020 and February 14, 2021, had their CXR severity quantified from 0 to 36 using the MBrixia score. Association between MBrixia score and received respiratory support was assessed by Poisson regression. Correlation between MBrixia score and blood biomarkers was assessed using Kendall rank correlation. Interobserver variability between three blinded radiologists was assessed by Kappa values and Bland-Altman plots. Associations between MBrixia score at admission and 90-day mortality were examined by adjusted Cox Proportional Hazards models. Among 279 patients with 606 CXRs, higher levels of respiratory support were associated with a higher estimated mean MBrixia score. Nine of 15 investigated blood biomarkers were significantly correlated with MBrixia score. Moderate agreement between radiologists was observed, with higher agreement among senior radiologists. Among 251 patients with CXRs at time of admission, all patients had an MBrixia score < 27. Those falling in the ranges, 10–18 and 19–27, compared to 0–9, had an adjusted hazard ratio for 90-day mortality of 1.22 (CI 95% 0.66–2.26) and 3.13 (CI 95% 1.26–7.76), respectively. The MBrixia score was validated as a marker of disease severity, and scores > 18 at admission were associated with a threefold increase in 90-day mortality.

**Supplementary Information:**

The online version contains supplementary material available at 10.1038/s41598-026-39285-x.

## Introduction

Chest x-ray (CXR) findings in COVID-19 patients include multilobed or bilateral lesions, often as ground glass opacities (GGOs) and consolidations^1^. GGOs frequently evolve into consolidations, peaking between day 6 to 11 in the disease course, and revert back to GGOs^2^. COVID-19 pneumonia can result in varying degrees of respiratory distress, ranging from mild cases to acute respiratory distress syndrome (ARDS)^3^.

Tools for quantifying the severity of pulmonary lesions in CXRs have shown potential as predictors of COVID-19 disease progression, clinical deterioration and mortality^4–14^, like the COVID-19 specific Brixia score, and the Radiographic Assessment of Lung Edema (RALE) score for evaluation of lung edema and percentage opacification^4,15^. Our group has previously published the MBrixia score, a modified Brixia score. The MBrixia score quantifies the severity of COVID-19 pneumonia in CXRs by dividing the lung (in an anteroposterior or posteroanterior projection) into 12 zones, as opposed to 6 zones used in the Brixia score. Each zone is given a score based on lesion severity, ranging from no lesions to consolidations^16^. In a population of patients admitted to a tertiary hospital in spring 2020, we found that the MBrixia score was associated with the clinical severity of COVID-19, measured by the received level of respiratory support at the time of CXR. Further, the MBrixia score correlated with several biomarkers^16^.

Various time points and methods for CXR-based score evaluation have been employed, including on-admission, minimum and maximum scores as well as score kinetics. Interestingly, several studies reported CXR-based scores at time of hospitalization to be predictive of mortality, but most reported from the first half of year 2020, and only few studies have studied the prognostic value of CXRs scores after the introduction of dexamethasone and remdesivir into standard of care treatment^7,14^.

The purpose of this study was to validate the findings of the previous study on the MBrixia score’s relation to disease severity and biomarkers, and to report on interobserver variability among radiologists applying the score. Further, we hypothesized that the MBrixia score at admission would be associated with mortality in a population with access to dexamethasone and remdesivir treatment options for COVID-19.

## Methods

### Study participants and source of data

Adult patients (≥ 18 years) admitted with a COVID-19 diagnosis to the department of infectious diseases or the intensive care unit (ICU) at our tertiary center, Rigshospitalet, Denmark, from June 15, 2020–February 14, 2021, who had undergone a CXR examination during hospitalization were included in this study. Patients admitted with a COVID-19 diagnosis at another hospital in Denmark and subsequently transferred to Rigshospitalet were included, using admission dates from initial admissions. In addition to the entire cohort outlined above, a subgroup of patients with a CXR available within two days after hospitalization were included in subgroup analysis focused on the prognostic value of CXRs at the time of admission *only.*

### Definitions

Data on respiratory support, demographics, comorbidities, prescribed medicine, biomarkers and the MBrixia scores were extracted from electronic healthcare records and PERSIMUNE^17^ with follow-up up to 26th of May 2021. Data elements were linked by social security numbers before pseudo anonymized extraction. Death dates were updated from the National Death Registry for complete follow-up.

The day of admission was defined as the first hospitalization day with a COVID-19 diagnosis. Comorbidity was defined as hypertension, diabetes mellitus, renal impairment, lung disease, or congestive heart failure according to previous studies^18–20^. Comorbidities were identified using diagnosis codes from the International Classification of Diseases, 10th edition (Specific codes are listed in Supplementary Table 1). Compromised immune function was defined as an immunosuppressive medical condition diagnosed before admission or receiving immunosuppressive treatment within six months prior to admission or recent corticosteroid treatment (detailed definition in Supplementary material). The level of respiratory support was defined on a four-level ordinal scale: receiving no supplemental oxygen, 1–5 liters (L) of oxygen/min, > 5 L oxygen/min, but not admitted to the ICU, and patients in the ICU, as previously described^16^. Due to a lack of data on the use of extracorporeal membrane oxygenation (ECMO), we were not able to differ between receiving invasive mechanical ventilation or ECMO in the ICU.

The primary outcome was death within 90 days from time of hospitalization with baseline time at day of hospitalization. An exploratory analysis of the outcome death within 90 days from maximum measured MBrixia score was also conducted.

### MBrixia scoring

The MBrixia score was defined according to our previously published study, by compartmentalizing and scoring a CXR in 12 anatomical zones spanning both lungs. Disease severity was scored on a scale from zero to three, producing an ordinal score variable in the range 0 to 36^18^. CXRs in the posteroanterior or anteroposterior projection were scored as part of the radiological routine at Rigshospitalet using the Radiology Information System/Picture Archiving and Communication Software System (RIS/PACS) from AGFA. CXRs were scored prospectively by resident radiologists supervised by senior or consultant radiologists, but data was not available on the radiologist experience for prospectively scored CXRs. The CXR imaging was performed at hospitals in the Capital Region of Denmark and Region Zealand. Different devices were used for imaging, depending on the hospital and whether a portable or non-portable device was used. Imaging procedures complied with national radiographic standards.

Several CXRs at time of admission were not scored during radiological routine for unknown reasons, and some were not scored due to patients transfers. Such CXRs were scored retrospectively by one of three radiologists with different experience (one resident, one senior, and one consultant) using Siemens Healthineers syngo.via software (Forchheim, Germany)^21^. To assess interobserver variability, we randomly chose 87 CXRs from the unscored CXRs to be scored by all three radiologists, who were blinded to referral text, patient outcomes and each other’s score. The 87 CXRs were scored in the same order by each radiologist. Based on extrapolations from a similar study, the sample size of 87 patients was chosen to observe mean differences between raters as small as 0.7 in MBrixia score, corresponding to 1.9% of MBrixia score range (0–36)^22^. The average of the 3 MBrixia scores for those CXRs was used in the mortality analysis.

### Statistical analysis

In the entire cohort, the association between the MBrixia score and the concurrent level of received respiratory support was tested by Poisson regression using generalized estimating equations with robust standard errors to account for multiple CXRs per patient. Incidence rate ratios and 95% confidence intervals (CI) were calculated using, “no supplemental oxygen,” as the reference group. The mean predicted MBrixia score at each level of respiratory support was estimated. Kendall rank correlation was used for correlation analysis of biomarkers and MBrixia score. To reduce the risk of type-1 errors in this analysis, a Bonferroni corrected p-value < 0.0033 for statistical significance was introduced. This method was identical to that used in our previous study^16^.

Interobserver variability was tested using Cohen’s linearly weighted κ and the scoring range was evaluated with Bland-Altman plots for comparing pairs. Fleiss’ linearly weighted κ was used for comparing three raters, using the Stata 17.0 “*kappaetc”* package^23^. Each κ-value was reported with a 95% CI.

### Mortality

In the *subgroup with available CXRs at time of admission*, the MBrixia score’s association with 90-day mortality was assessed in uni- and multivariable Cox regression analyses and using Kaplan-Meier plots. All patients were followed for 90 days from time of hospitalization. The MBrixia score variable was handled both as a numerical variable and as a categorical variable, in two separate analyses. The categorical variable was created by dividing the total score range into four equally distributed intervals (“0–9”, “10–18”, “19–27” and “28–36”) as extrapolated by the study by Au-Yong et al.^6^. The following predefined variables were added to the multivariable models: “age per 10 years”, “sex”, “comorbidity”, “immunocompromised from preexisting medical condition or medication”. Using the *entire cohort*, we assessed the association between the maximum in-hospital MBrixia score and 90-day mortality using the same method but using the time of maximum MBrixia score as baseline time.

## Results

### Study population

**Fig. 1:**
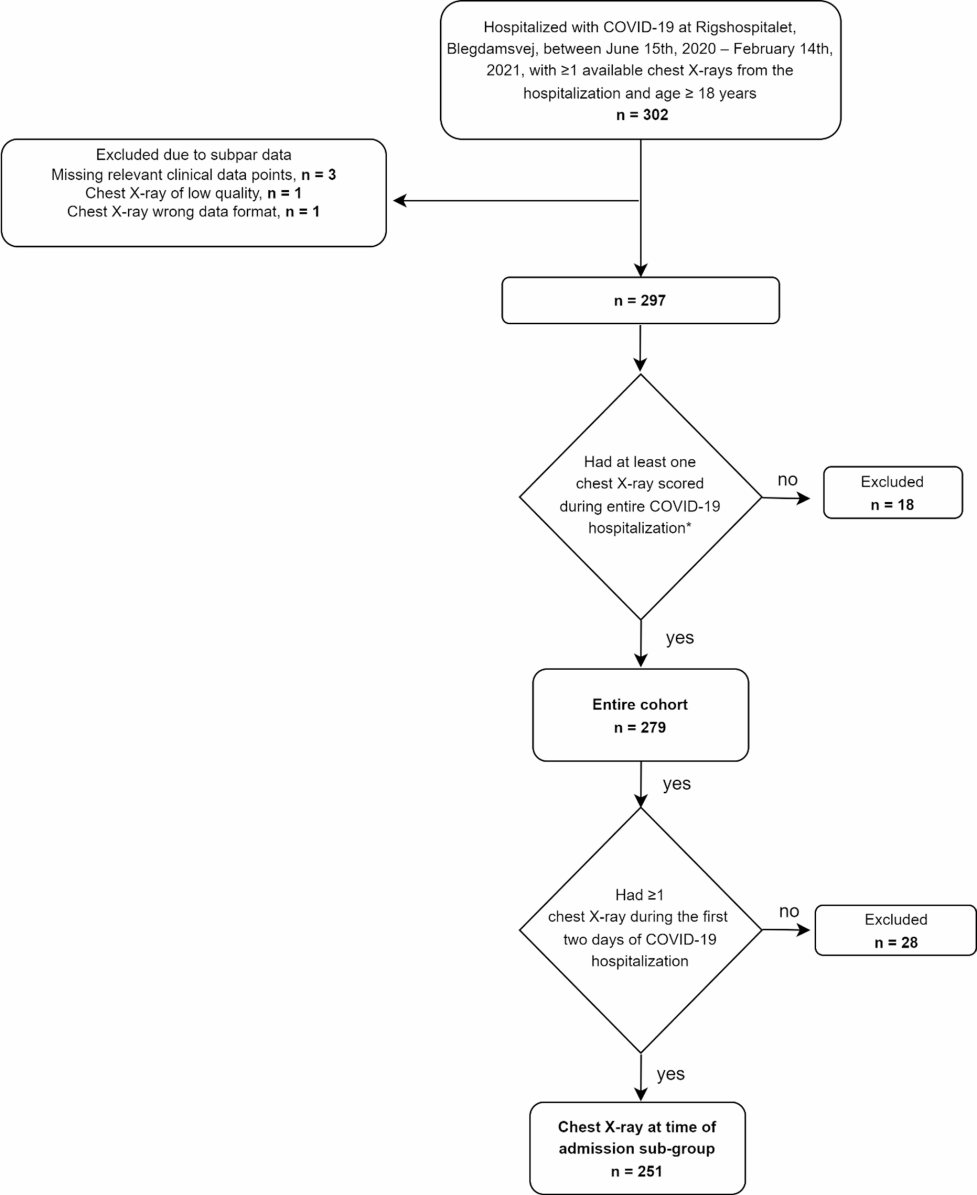
Flow chart describing the inclusion criteria for the subgroup with at admission CXRs and the entire cohort. Of the 302 identified patients, 5 were excluded due to subpar data. Of the remaining 297 patients, 251 patients had chest X-rays performed in the first two days of their COVID-19 hospitalization that were scored prospectively or retrospectively to achieve a complete data set. Since only CXRs from the first two days of hospitalization were scored retrospectively, an amount of CXRs was not scored in the entire cohort, leading to 18 excluded patients that did not have a CXR in the first 2 days of hospitalization but did have a later unscored CXR.

Of the 302 patients identified, 279 were included in the entire cohort of which 251 were in the subgroup with available CXRs at time of admission (Fig. 1). Baseline characteristics for both groups are presented in Table 1. Patients were predominantly male (62.4% in the entire cohort and 61.0% in the subgroup). The median age was 66 years and a high proportion of patients in both the entire cohort and the subgroup had comorbidities. The subgroup had similar baseline characteristics as the entire cohort. Patients excluded had a lower proportion of comorbidities (Supplementary Table 2). Prospectively scored CXRs were available for 179 (64.2%) patients in the entire cohort. Of the 251 patients in the subgroup, 86 (34.3%) had a prospectively scored CXR at admission; the remaining patients‘ CXR from time of admission was scored retrospectively (165 CXRs [65.7%]). At admission, no patients had an MBrixia score in the range “28–36”. Of the 877 CXRs performed during hospitalization in the entire cohort, 608 (69,3%) were scored. Of the 608 CXRs in the entire cohort, 443 (72,9%) were scored prospectively. Within 90 days of hospitalization, 58 (20.8%) patients died in the entire cohort, of which 53 (21.1%) died within the subgroup. No patients were lost to follow-up.

**Table 1:** Baseline characteristics of patients included in the entire cohort and subgroup with at admission CXRs.

Patient characteristics	CXR at time of admission subgroup	Entire cohort
Number of patients (n)	251	279
Sex, n (%)		
Male	153 (61.0%)	174 (62.4%)
Female	98 (39.0%)	105 (37.6%)
Age at admission, median (IQR)	66.0 (56.0–76.0)	66.0 (56.0–76.0)
MBrixia score at admission, median (IQR)	11.7 (7.0-15.3)	N/A
Congestive heart failure, (%)	25 (10.0%)	25 (9.0%)
Chronic pulmonary disease, n (%)	39 (15.5%)	41 (14.7%)
Diabetes mellitus, n (%)	46 (18.3%)	49 (17.6%)
Chronic renal disease, n (%)	27 (10.8%)	32 (11.5%)
Hypertensive disease, n (%)	54 (21.5%)	57 (20.4%)
Any of the described comorbidities, n (%)	116 (46.2%)	125 (44.8%)
Immunocompromised due to condition or medication, n (%)	75 (29.9%)	85 (30.5%)

### Interobserver variability

**Fig. 2:**
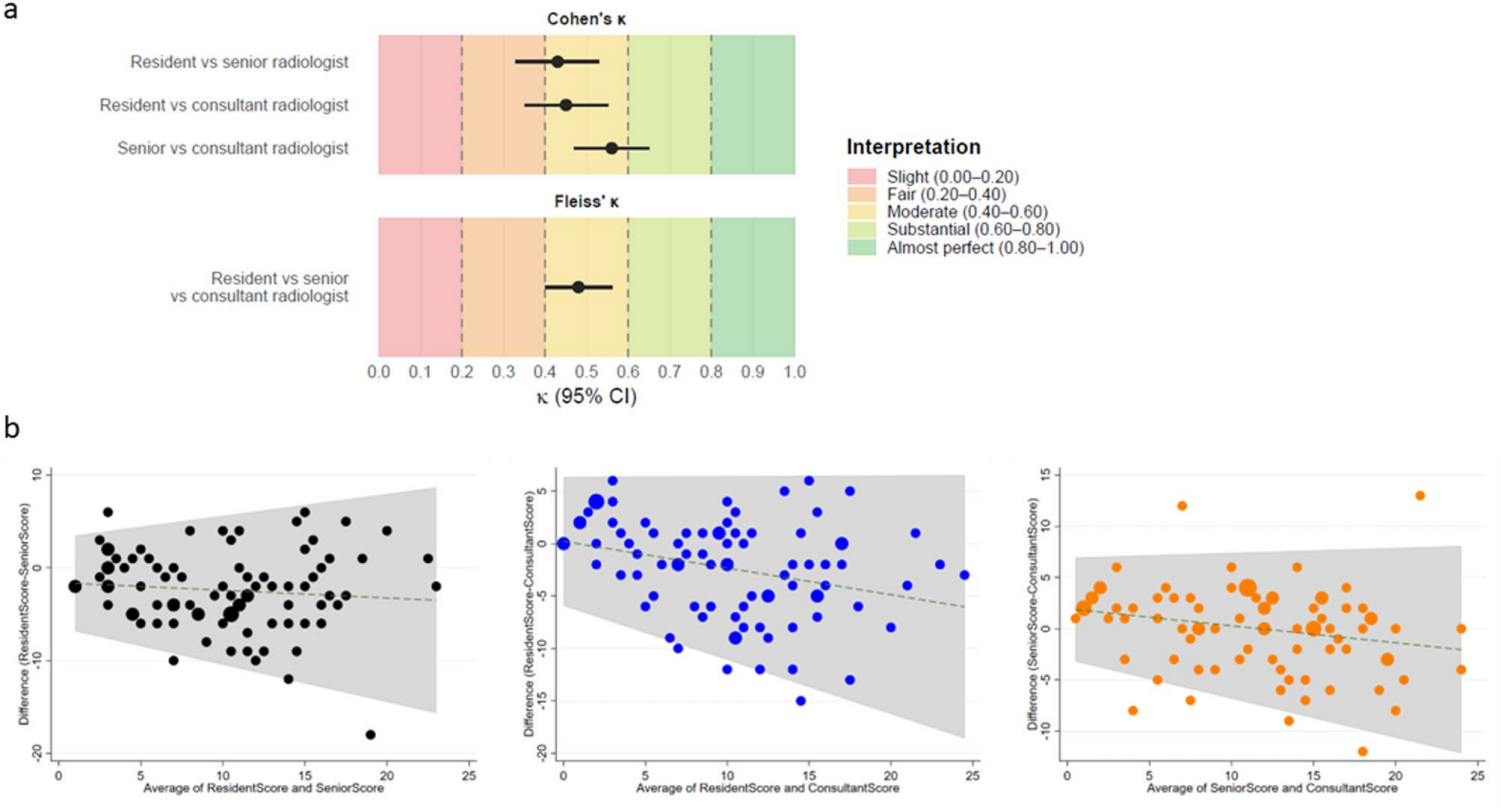
(**a**) Interobserver variability among three radiologists assessing 86 chest X-rays by the MBrixia score algorithm assessed by Cohen’s linearly weighted κ for comparing pairs of radiologists with different experience and Fleiss’ linearly weighted κ for comparing three raters at once. The zones of κ depicted in the background of the plot are as defined by Landis and Koch, 1977, and (**b**) Bland-Altman plots for 86 chest x-rays assessed by the MBrixia score algorithm from left: resident radiologist versus a senior radiologist, resident radiologist versus a consultant radiologist and senior radiologist versus a consultant radiologist..

Interobserver variability was assessed in 86 randomly chosen CXRs from time of admission, all scored retrospectively by three radiologists. One CXR was excluded due to poor image quality. All comparisons among radiologists resulted in moderate agreement (Fleiss’ κ 0.48 (95% CI 0.40–0.56)) according to the Landis and Kochs definition (Fig. 2a)^24^. Agreement between experienced radiologists (senior and consultant radiologist) was higher (Cohen’s κ 0.56 (95% CI 0.47–0.65)) compared to agreement between the resident and the senior radiologist (Cohen’s κ 0.43 (95% CI 0.33–0.53)), and the consultant radiologist (Cohen’s κ 0.45 (95% CI 0.35–0.55)), however, these differences were not statistically significant. No differences were found when stratifying the analysis into the first 43 rated vs. the last 43 rated CXRs (Supplementary Table 3). Bland-Altman plots suggested that disagreement between raters increased with higher average MBrixia scores, and a trend of higher scoring by the more experienced radiologist was observed (Fig. 2b).

### The MBrixia score and biomarkers correlation

In the entire cohort, MBrixia score was tested for correlation with 15 time-matched biomarkers among 608 scored CXRs (Supplementary Fig. 1a). We found statistically significant correlations between the MBrixia score and neutrophil count, eosinophil count, fibrin D-dimer, C-reactive protein (CRP), alanine aminotransferase (ALT), plasma ferritin and lactate dehydrogenase (LDH) (all *p* < 0.001). In addition, we found hemoglobin and plasma albumin to be inversely correlated with MBrixia (both *p* < 0.001). There was a correlation trend towards thrombocyte count (*p* = 0.045) and procalcitonin (*p* = 0.038), but estimates did not reach Bonferroni correction significance threshold (p-value < 0.0033) (Supplementary Table 4).

### The MBrixia score and level of respiratory support

Compared to patients receiving no supplemental oxygen, those receiving 1–5 L oxygen/min, > 5 L oxygen/min, and patients in the ICU had a 1.51 (95% CI 1.35–1.69, *p* < 0.0001), 1.83 (1.62–2.06, *p* < 0.0001), and 2.24 (1.99–2.53, *p* < 0.0001) times higher estimated mean MBrixia, respectively. Similarly, the mean predicted MBrixia scores of patients at each level of received respiratory support were 9.37 (95% CI 8.46–10.3), 14.14 (13.25–15.09), 17.16 (15.97–18.42) and 21.02 (19.72–22.40), respectively (Supplementary Fig. 1b).

### The MBrixia score at admission and its association with 90-day mortality

In this subgroup analysis, the association between MBrixia score at the time of hospitalization and death within 90 days of hospitalization (90-day mortality) was assessed by Cox proportional-hazards models and Kaplan Meier plots (Fig. 3a). In univariable analysis, MBrixia score at admission, fitted either as a numerical or as a categorial variable was not associated with mortality, but age and the existence of comorbidities were associated with an increased risk of death. However, in multivariable analyses, an MBrixia score within range “19–27”, was associated with a three times higher rate of mortality within 90 days (aHR 3.13, CI 95%: 1.26–7.76) (Fig. 3b) compared to those with a score of “0–9”. Additionally, the association remained when using MBrixia score as a numerical variable in the otherwise identically adjusted model (aHR 1.06 [95% CI 1.00–1.11] *p* = 0.04) (Supplementary Table 5).

Multiple sensitivity analyses were performed. When adjusting for level of respiratory support, MBrixia score remained significantly associated with 90-day mortality, despite likely collinearity between the two variables (Supplementary Table 6). When substituting the MBrixia score with level of respiratory support in multivariable analysis, receiving > 5 L O2/minute was borderline associated with 90-day mortality, while being in the ICU was not. This may be due to confounding by indication, as patients with severe COVID-19 but too frail to be transferred to the ICU may land in the “receiving > 5 L O2/minute” category instead (Supplementary Table 7). When excluding patients transferred to the tertiary referral hospital, MBrixia score remained associated with 90-day mortality (Supplementary Table 8). Lastly, we assessed the association between 90-day mortality and the maximum in-hospital MBrixia score in the entire cohort. In univariable analysis, maximum MBrixia score assessed as a numerical variable was associated with 90-day mortality since maximum MBrixia score (crude HR 1.06 [95% CI 1.03–1.10]). When assessed as a categorical variable, the score-range “19–27” was borderline significant (cHR 2.20 [95% CI 0.99–4.80]) and the maximum range “28–36” was significantly associated with 90-day mortality (cHR 5.28 [95% CI 1.91–14.54]). In multivariable analysis, a maximum MBrixia score within ranges “19–27” and “28–36” were associated with an increased risk of death within 90 days (Supplementary Fig. 2).

**Figure:**
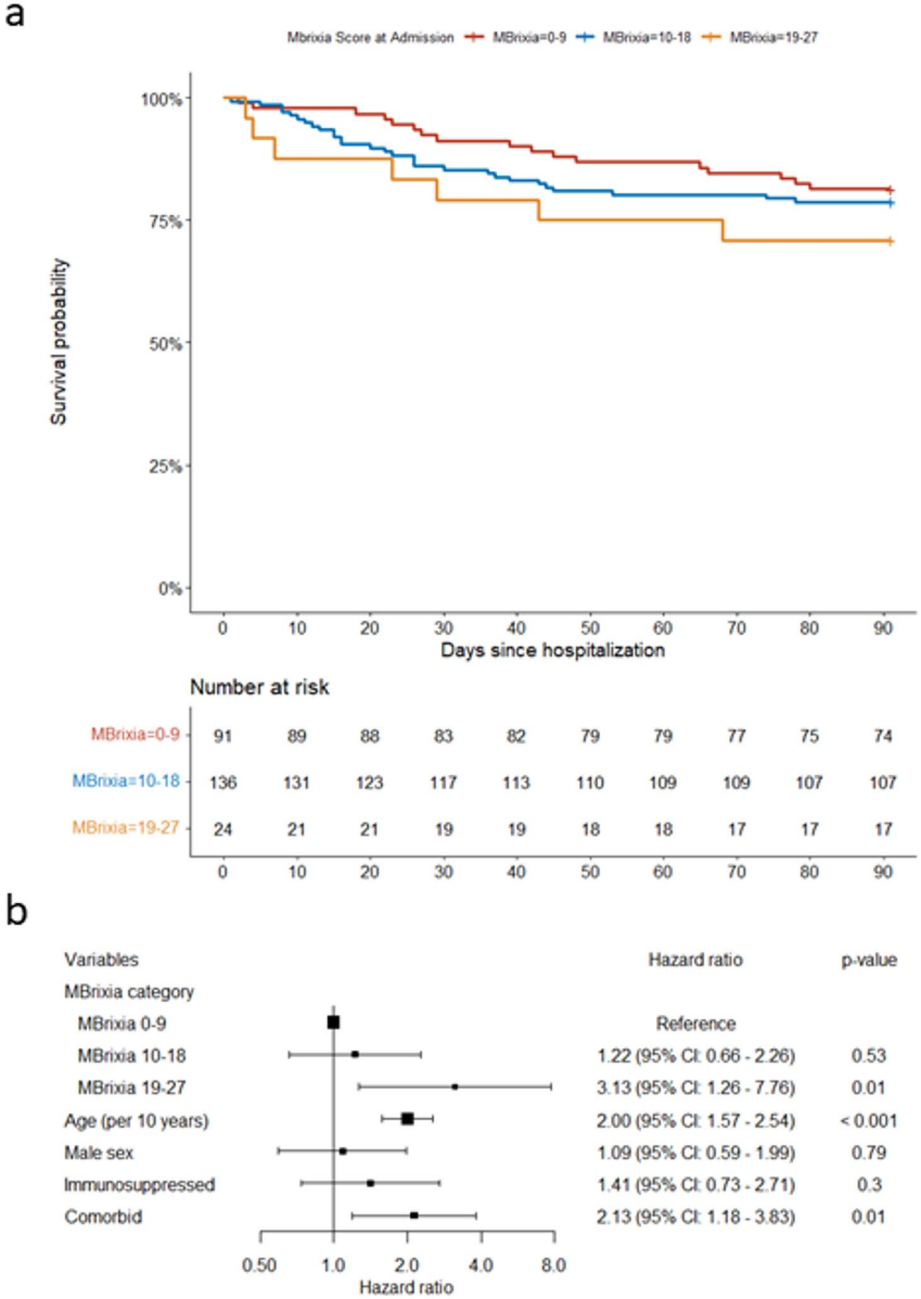
(**a**) Kaplan-Meier plot of the survival probability for three distinct curves representing the ranges 0–9, 10–18, and 18–27 of the MBrixia score at time of admission (no chest X-rays were scored higher than 27). Data was censored at 90 days after hospitalization. (**b**) Forest plot of hazard ratios for MBrixia score range on time of admission and risk factors from a multivariable Cox proportional hazards model for 90-day mortality since hospitalization.

## Discussion

We used the MBrixia score to quantify COVID-19 lung lesions and found that a high MBrixia score at the time of admission was associated with 90-day mortality. We further validated our group’s previously reported findings on the MBrixia score association with disease severity using level of received respiratory support and blood biomarkers as by-proxy indicators. Lastly, we assessed interobserver variability, and found moderate agreement among radiologists at different levels of experience applying the MBrixia score to CXRs.

The MBrixia score’s incremental relation with an increased level of respiratory support was validated, apart from that ECMO patients were not identifiable in our dataset^16^. Also, correlation analyses between the MBrixia score and time-matched blood biomarkers were validated for most of the investigated biomarkers. Among the leukocytes, elevated neutrophiles and eosinophiles were correlated with the MBrixia score. While neutrophilia has been reported extensively to be associated with severe COVID-19 disease, eosinopenia has been found to be associated with lower survival in some studies, however other studies have showed that secondary eosinophilia developed during admission was associated with increased morbidity in critically ill COVID-19 patients^25,26^. Eosinophil-related complications have been reported in COVID-19, including treatment-associated hypersensitivity reactions and eosinophil-driven conditions. Longitudinal immunologic studies in severe COVID-19 have also demonstrated activation of type 2 inflammatory pathways (IL-4, IL-5, IL-13) associated with worse clinical outcomes^27,28^.

The acute phase reactants fibrin D-dimer, ferritin, LDH and CRP, as well as and albumin and hemoglobin are well known to be associated positively or inversely with increased COVID-19 disease severity^29–34^. Our validated findings emphasize the robustness of using the MBrixia score as a by-proxy measure of disease severity derived from CXR pulmonary lesions. We did, however, find contradicting results with the initial smaller pilot study for ALT, hemoglobin, monocyte count and thrombocyte counts.

The interobserver variability agreement on the MBrixia score was moderate, regardless of being scored by a skilled thoracic radiologists or radiologists undergoing training at different levels of experience. A higher level of agreement was reported for both the Brixia score and the RALE score in a previous study^15^, but those scores also have fewer zones to score. We saw trends in the analyses towards an increased score variability at higher MBrixia scores, as well as a higher level of agreement among the two radiologists with most experience.

Our findings on interobserver variability emphasize an important limitation of human-derived CXR scoring and highlight the challenges of relying on manual assessment in clinical workflows. In this context, AI-model-based CXR score generation, such as the MBrixia score, could be valuable to scale the scoring process in the pandemic scenario where resources may be limited and ultimately limit variability in scoring. However, to learn such models MBrixia scoring by supervised learning, their performance is fundamentally constrained by the quality, consistency, and reproducibility of the human-produced reference labels on which they are trained. Interobserver variation therefore not only affects bedside scoring but may also be propagated into the models themselves. Therefore large, curated datasets of CXRs scored with the MBrixia score by experienced radiologists would be advised for such effort. An alternative approach is to train models directly from CXR images to the same clinical outcomes, however this approach may reduce the interpretability and produce “black box” predictions with limited insight into the radiographic features driving a given output and thereby making it difficult for clinicians to oversee and ultimately trust predictions, if not made explainable. Regardless of AI or human based CXR assessments, both approaches require a robust external validation to demonstrate generalizability across diverse geographic, socioeconomic, and clinical settings.

The MBrixia score examined at admission was associated with 90-day mortality in adjusted Cox proportional hazards models and MBrixia score was associated with disease severity measured by respiratory support. The observed association with disease severity is consistent with our prior report showing that higher levels of respiratory support required during admission are associated with lower recovery rates^18^. Both studies took place after dexamethasone and remdesivir treatment became standard of care, therefore suggesting that CXR scores at time of admission were still prognostic for mortality. These findings are in line with those reported on the Brixia score by Borghesi et al. from the second COVID-19 wave, despite the improved survival rates during the second wave^5,14,18,35–37^. The time of admission is a comparable, robust, and generalizable timepoint and creates an opportunity for intervention. The score could possibly be optimized further in detecting GGOs for better predictive capabilities at time of admission, since GGOs present earlier in the disease and later may develop into consolidations.

This study has several limitations. First, none of the patients had an MBrixia score > 27 at admission, although higher scores have been observed later during hospitalization and have been associated with disease severity. Second, inclusion of patients transferred from other hospitals may have introduced immortal time bias. However, because patients were identified retrospectively from routine clinical data, we expect selection bias to be limited. Third, the cohort included a high proportion of immunocompromised patients and was drawn from a tertiary referral hospital in the Capital Region, which may limit representativeness compared with the background population. Therefore, the score should be evaluated in broader, non-tertiary settings to validate generalizability. Fourth, a prospective study with predefined timepoints for CXRs would enable more precise characterization of longitudinal score trajectories, but it may increase radiation exposure and may not be feasible or ethically justified in all settings. Finally, the cohort is historical, and the findings are not directly comparable with the contemporary clinical landscape of COVID-19 in a vaccinated population and with newer drugs available. Nevertheless, because the score captures the spatial extent of lung involvement, it may retain relevance as a general marker of severity and still be able to discriminate future risk despite new interventions lowering the overall risk of COVID-19 related morbidity and mortality.

## Conclusion

The MBrixia score was validated as being associated with COVID-19 disease severity and correlated with levels of a range of blood biomarkers. Higher MBrixia scores at admission were associated with 90-day mortality. Interrater variability analyses demonstrated moderate agreement despite differences in seniority among the radiologists. These findings suggest that CXR-based scoring tools such as the MBrixia score are easily available and provide a quantification that can be applied in both clinical and research settings to characterize the course of COVID-19 pneumonia. However, contemporary data will be required to assess predictive performance in populations with high vaccine coverage and in the context of emerging SARS-CoV-2 variants.

## Supplementary Information

Below is the link to the electronic supplementary material.

Supplementary Material 1

## Data Availability

Data available from the corresponding author upon reasonable request.
