# Supplementary material for: Early chest x-ray severity assessed by the MBrixia score is associated with mortality in hospitalized patients with COVID-19 pneumonia
Source: Sci Rep. 2026 Feb 11;16:8309. doi: 10.1038/s41598-026-39285-x (PMC12966410; doi:10.1038/s41598-026-39285-x)

Supplementary Material

Methods

Immunocompromised definition

The definition of immunocompromised due to use of corticosteroids was treatment of more than five mg dexamethasone or another corticosteroid with an equivalent dose of > 5 mg dexamethasone, for more than one week, within a month prior to hospitalization date. Treatment was defined using the Anatomical Therapeutic Chemical Classification System (ATC) using the following ATC-codes: H02, L01 and L04. For corticosteroids specifically (H02), patients were categorized as immunocompromised if having received oral or intravenous therapy. For medical conditions, all conditions present in the cohort were manually screened by physicians CMJ and KSM, and a list of immunocompromising conditions were compiled.

Software

Statistical analyses were done in Stata 17.0 (StataCorp, College Station, Texas, USA), SAS Studio 9.4 (SAS Institute Inc., Ca,ry, NC, USA) and R 3.6.1 (R Core Team).

Supplementary Table 1: Comorbidity definition

Supplementary Table 1: ICD-10 codes used for defining comorbidities. All ICD-10 codes with the mentioned prefix are included.

Comorbidity	ICD-10 codes used
**Chronic heart failure**	I21 I22 I252 I43 I50 I425 I426 I427 I428 I429 I099 I110 I130 I132 I255 I420 P290
**Chronic lung disease**	J40 J41 J42 J43 J44 J45 J46 J47 J60 J61 J62 J63 J64 J65 J66 J67 I278 I279 J684 J701 J703
**Diabetes mellitus**	E100 E101 E106 E108 E109 E110 E111 E116 E118 E119 E120 E121 E126 E128 E129 E130 E131 E136 E138 E139 E140 E141 E146 E148 E149 E102 E103 E104 E105 E112 E113 E114 E115 E122 E123 E124 E125 E132 E133 E134 E135 E142 E143 E144 E145 E107 E117 E127 E137 E147
**Chronic renal disease**	N18 N19 N032 N033 N034 N035 N036 N037 N052 N053 N054 N055 N056 N057 Z490 Z491 Z492 I120 I131 N250 Z940 Z992
**Hypertension**	I1

Results

Supplementary Table 2: Baseline characteristics extended

Supplementary Table 2 Baseline characteristics of excluded and included patients in both entire cohort and in the subgroup with at admission CXRs.

Characteristics	Entire cohort		Subgroup with at admission CXRs	
	**Excluded**	**Included**	**Excluded**	**Included**
**Count**	n = 23	n = 279	n = 51	n = 251
**Sex, count (%)**				

**Male**	12 (52.2%)	174 (62.4%)	33 (64.7%)	153 (61.0%)
**Female**	11 (47.8%)	105 (37.6%)	18 (35.3%)	98 (39.0%)
**Age at admission, median (IQR)**	66.0 (53.0-71.0)	66.0 (56.0-76.0)	65.0 (55.0 -73.0)	66.0 (56.0-76.0)
**MBrixia score at admission, median (IQR)**	N/A	N/A	N/A	12.0 (7.0-16.0)
**Congestive heart failure, count (%)**	<5 (<21.7%)	25 (9.0%)	<5 (<9.8%)	25 (10.0%)
**Chronic pulmonary disease, count (%)**	<5 (<21.7%)	41 (14.7%)	<5 (<9.8%)	39 (15.5%)
**Diabetes mellitus, count (%)**	<5 (<21.7%)	49 (17.6%)	6 (11.8%)	46 (18.3%)
**Chronic renal disease, count (%)**	<5 (<21.7%)	32 (11.5%)	6 (11.8%)	27 (10.8%)
**Hypertensive disease, count (%)**	<5 (<21.7%)	57 (20.4%)	5 (9.8%)	54 (21.5%)
**Any of described comorbidities, count (%)**	5 (21.7%)	125 (44.8%)	14 (27.5%)	116 (46.2%)
**Immunocompromised due to condition or medication, count (%)**	6 (26.1%)	85 (30.5%)	16 (31.4%)	75 (29.9%)
**Dead within 90 days of admission, count (%)**	<5 (<21.7%)	58 (20.8%)	8 (15.7%)	53 (21.1%)

Supplementary Table 3: Interrater variability

Supplementary Table 3 Assessment of interrater variability and estimated Kappa values between pairs (Cohen's κ) and all raters (Fleiss' *κ)*

	**All CXRs**	**First scored chest X-rays**	**Last scored chest X-rays**
**Cohen's κ (95% CI)**			
**Resident vs senior radiologist**	0.43 (0.33 – 0.53)	0.40 (0.24 – 0.56)	0.45 (0.31 – 0.59)
**Resident vs consultant radiologist**	0.45 (0.35 – 0.55)	0.47 (0.33 – 0.60)	0.44 (0.29 – 0.60)
**Senior vs consultant radiologist**	0.56 (0.47 – 0.65)	0.57 (0.44 – 0.71)	0.55 (0.43 – 0.66)
**Fleiss' κ (95% CI)**			
**Resident vs senior vs consultant radiologist**	0.48 (0.40 – 0.56)	0.48 (0.36 – 0.60)	0.47 (0.34 – 0.60)

Supplementary Table 4: Correlation with biomarkers from blood tests, data behind figure

Supplementary Table 4 Kendall rank correlation coefficient analysis of MBrixia score from CXR and 15 time-matched biomarker analyses measured as part of routine care.

**Biomarker from blood tests**	**Observations (n)**	**Missing values from time matched definiton**	**tau-b**	**p-value**
**Alanine aminotransferase**	588	20	0.127	<.001*
**Creatinine**	607	1	0.003	0.907
**C-reactive protein**	606	2	0.103	<.001*
**Eosinophil count**	447	161	0.152	<.001*

**Fibrin d-dimer**	360	248	0.390	<.001*
**Fibrinogen**	136	472	-0.077	0.197
**Hemoglobin**	608	0	-0.281	<.001*
**Lactate dehydrogenase**	570	38	0.253	<.001*
**Lymphocyte count**	449	159	0.018	0.582
**Monocyte count**	447	161	0.044	0.177
**Neutrophil count**	449	159	0.244	<.001*
**Plasma albumin**	504	104	-0.191	<.001*
**Plasma ferritin**	447	161	0.127	<.001*
**Procalcitonin**	255	353	0.090	0.038
**Thrombocyte count**	605	3	0.056	0.045

*Statistically significant (Bonferroni adjusted p-value < .0033)

Supplementary Table 5: Assessing mortality with uni- and multivariable Cox regression

Supplementary Table 4: Cox model data behind forest plots in the main manuscript. MBrixia score tested as both tertiles and continuous variable.

			**MBrixia score as quartiles**		**MBrixia score as continuous variable**	
	**Crude HR (95% CI)**	**p-value**	**Adjusted HR (95% CI)**	**p-value**	**Adjusted HR (95% CI)**	**p-value**
**On-admission MBrixia score (numerical)**	1.03 (.98 – 1.08)	.26	-	-	**1.06 (1.00 – 1.11)***	**.04**
**0-9**	Ref.	-	Ref.	-	-	-
**10-18**	1.20 (.66 – 2.19)	.55	1.22 (.66 – 2.26)	.53	-	-
**19-27**	1.75 (.73 – 4.22)	.21	**3.13 (1.26 – 7.76)***	**.01**	-	-
**28-36**	-	-	-	-	-	-
**Age, per 10 years**	**1.91 (1.54 – 2.37)***	**<.01**	**2.00 (1.57 – 2.54)***	**<.01**	**1.97 (1.55 – 2.50)***	**<.01**
**Male sex**	1.26 (.71 - 2.22)	.43	1.09 (.59 – 1.99)	.79	1.22 (.67 – 2.22)	.51
**Comorbidity**	**2.72 (1.53 – 4.85)***	**<.01**	**2.13 (1.18 – 3.83)***	**.01**	**2.24 (1.25 – 4.01)***	**<.01**
**Immunocompromised**	.98 (.54 – 1.76)	.94	1.41 (.73 – 2.71)	.30	1.25 (.66 – 2.34)	.50

*** p < .05**

Supplementary Table 6: Cox model assessing 90-day mortality, including level of respiratory support

	Adjusted HR (95% CI)	p-value
On-admission MBrixia score		
0-9	Ref.	-
10-18	1.24 (.64 – 2.39)	.53
19-27	2.70 (0.99 –7.34)	.05
28-36	-	-
Age, per 10 years	2.03 (1.59 – 2.60)*	<.01
Male sex	.96 (.52 – 1.78)	.90
Comorbidity	2.23 (1.23 – 4.05)*	.01
Immunocompromised	1.36 (.71 – 2.62)	.35
Respiratory support		
No O2	Ref.	-
1-5 L O2/minute	.80 (.42 – 1.53)	.50
>5 L O2/minute	1.68 (.42 – 4.50)	.31
In the ICU	1.33 (.44 – 3.99)	.62

Supplementary Table 7: Cox model assessing 90-day mortality, substituting MBrixia score with level of respiratory support

	Adjusted HR (95% CI)	p-value
Age, per 10 years	1.97 (1.55 – 2.50)*	<.01
Male sex	.94 (.51 – 1.72)	.84
Comorbidity	2.27 (1.26 – 4.10)*	.01
Immunocompromised	1.24 (.65 – 2.34)	.51
Level of respiratory support		
No O2	Ref.	-
1-5 L O2/minute	.89 (.48 – 1.64)	.70
>5 L O2/minute	2.36 (.95 – 5.86)	.06
In the ICU	1.57 (.53 – 4.65)	.41

Supplementary Table 8: Cox model assessing 90-day mortality, excluding transferred patients (n = 125)

	Adjusted HR (95% CI)	p-value
On-admission MBrixia score		
0-9	Ref.	-
10-18	1.37 (.54 – 3.48)	.51
19-27	6.80 (1.59 –29.03)	.01
28-36	-	-
Age, per 10 years	2.45 (1.62 – 3.70)*	<.01
Male sex	.87 (.32 – 2.36)	.90
Comorbidity	3.00 (1.20 – 7.53)*	.02
Immunocompromised	2.00 (.72 – 5.59)	.18
Level of respiratory support		
No O2	Ref.	-
1-5 L O2/minute	.80 (.42 – 1.53)	.50
>5 L O2/minute	1.68 (.42 – 4.50)	.31
In the ICU	1.33 (.44 – 3.99)	.62

Supplementary Figure 1: Validation analyses

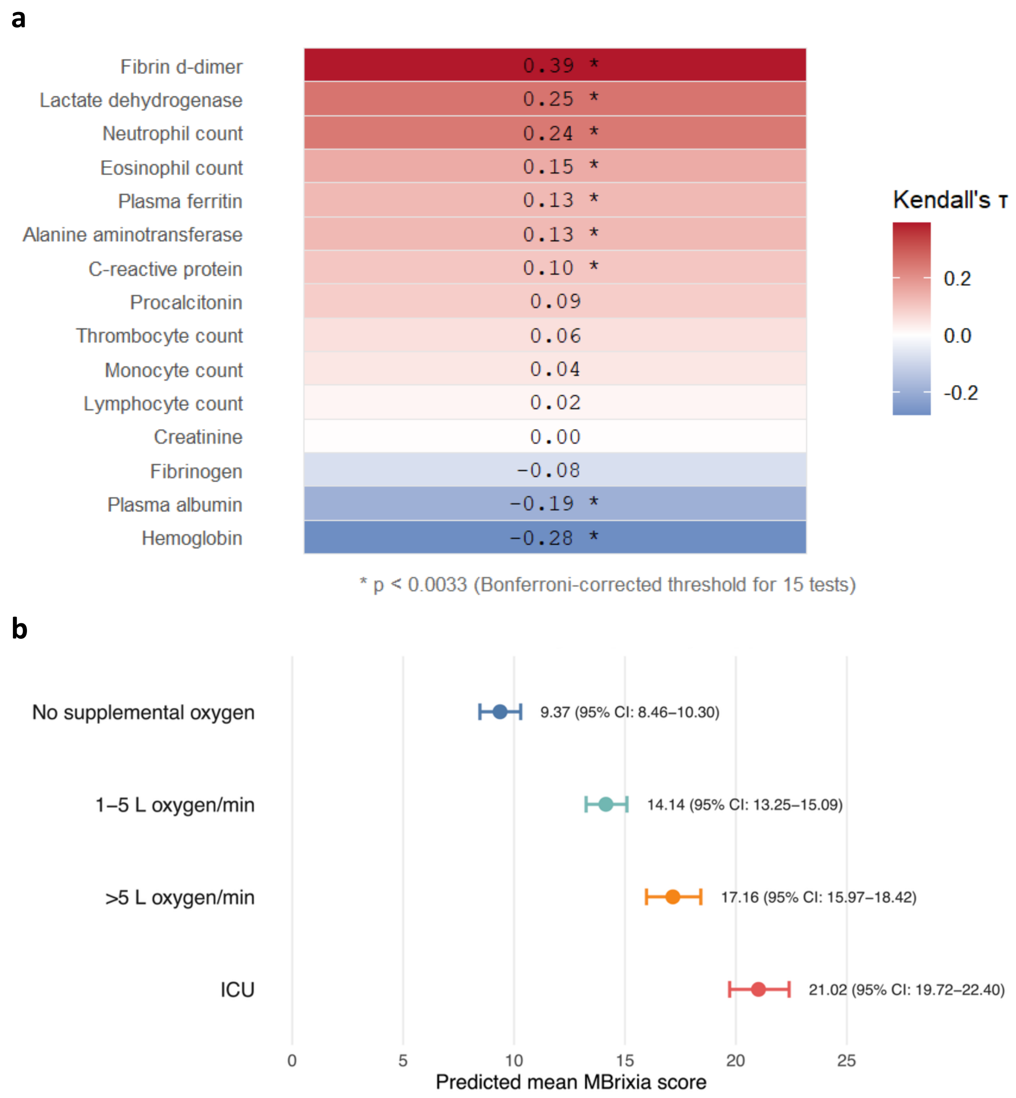

Supplementary figure 1 A) Estimated Tau-B, with Kendall's rank correlation, between time matched MBrixia score and 15 biomarkers (fibrin D-dimer, lactate dehydrogenase, neutrophil count, eosinophil count, thrombocyte count, lymphocyte count, monocyte count, creatine, fibrinogen, plasma albumin, hemoglobin, plasma ferritin, alanine aminotransferase, C-reactive protein and procalcitonin). Correlations that reached a Bonferroni corrected significance threshold of $p < 0.0033$ are marked with "*". B) The predicted mean MBrixia score among predefined levels of respiratory support at time of chest X-ray. Tested by Poisson regression using generalized estimating equations with robust standard errors to account for potential bias caused by patients having multiple chest X-rays performed.

Supplementary Figure 2: Max MBrixia score

a Survival Since Chest X-ray with Maximum MBrixia Score in Entire Admission Among All Patients

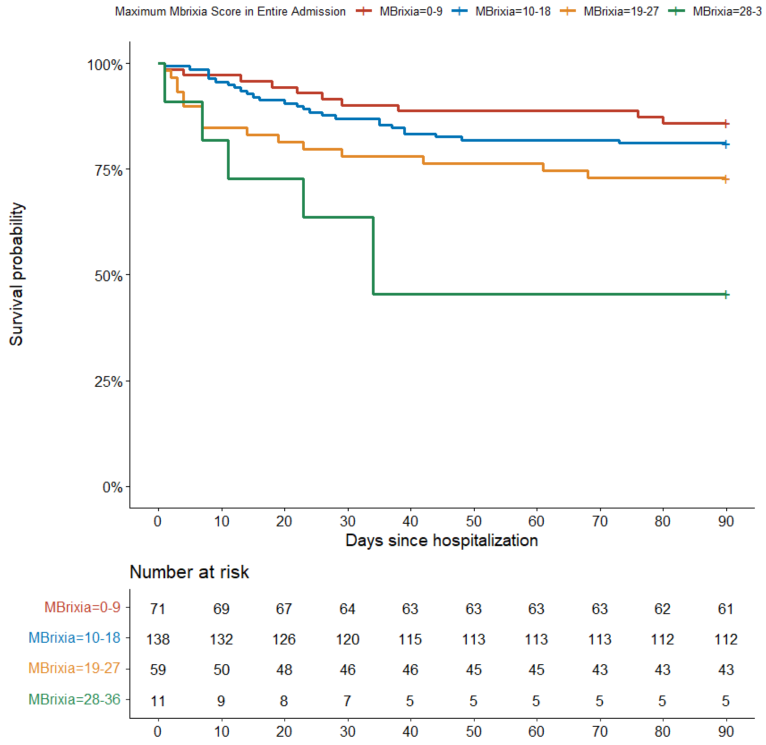

b 90-day Mortality Since Chest X-ray with Maximum MBrixia score in Entire Admission Among All Patients: Cox Proportional-Hazards Model

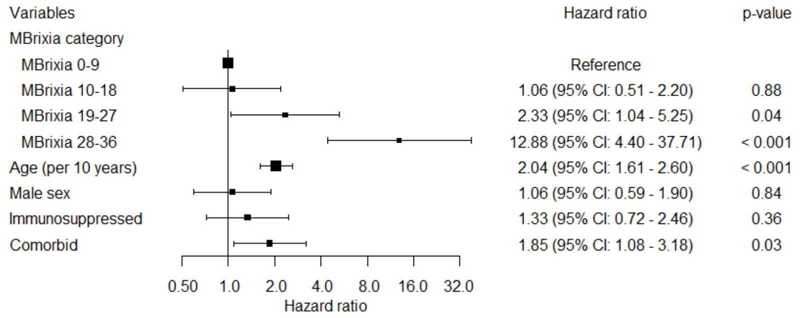

Supplement: Supplementary file 1 — Supplementary Material 1 [file 41598_2026_39285_MOESM1_ESM.pdf]
